# Characteristic Male Urine Microbiomes Associate with Asymptomatic Sexually Transmitted Infection

**DOI:** 10.1371/journal.pone.0014116

**Published:** 2010-11-24

**Authors:** David E. Nelson, Barbara Van Der Pol, Qunfeng Dong, Kashi V. Revanna, Baochang Fan, Shraddha Easwaran, Erica Sodergren, George M. Weinstock, Lixia Diao, J. Dennis Fortenberry

**Affiliations:** 1 Department of Biology, Indiana University, Bloomington, Indiana, United States of America; 2 Division of Infectious Diseases, Department of Medicine, Indiana University School of Medicine, Indianapolis, Indiana, United States of America; 3 Department of Biological Sciences, University of North Texas, Denton, Texas, United States of America; 4 Department of Computer Science and Engineering, University of North Texas, Denton, Texas, United States of America; 5 Department of Genetics, Washington University St. Louis School of Medicine, St. Louis, Missouri, United States of America; 6 Department of Bioinformatic and Computational Biology, University of Texas, M.D. Anderson Cancer Center, Houston, Texas, United States of America; 7 Section of Adolescent Medicine, Department of Pediatrics, Indiana University School of Medicine, Indianapolis, Indiana, United States of America; Duke University, United States of America

## Abstract

**Background:**

The microbiome of the male urogenital tract is poorly described but it has been suggested that bacterial colonization of the male urethra might impact risk of sexually transmitted infection (STI). Previous cultivation-dependent studies showed that a variety of non-pathogenic bacteria colonize the urethra but did not thoroughly characterize these microbiomes or establish links between the compositions of urethral microbiomes and STI.

**Methodology/Findings:**

Here, we used 16S rRNA PCR and sequencing to identify bacteria in urine specimens collected from men who lacked symptoms of urethral inflammation but who differed in status for STI. All of the urine samples contained multiple bacterial genera and many contained taxa that colonize the human vagina. Uncultivated bacteria associated with female genital tract pathology were abundant in specimens from men who had STI.

**Conclusions:**

Urine microbiomes from men with STI were dominated by fastidious, anaerobic and uncultivated bacteria. The same taxa were rare in STI negative individuals. Our findings suggest that the composition of male urine microbiomes is related to STI.

## Introduction

Sequencing of bacterial 16S rRNA alleles from the human skin, mouth, gastrointestinal tract and vagina has revealed that each of these sites supports surprisingly diverse microbial communities, including substantial numbers of uncharacterized and uncultivated species [1], [2]. These microbial communities are believed to play a key role in maintaining health, and altered microbiomes are associated with a variety of conditions including obesity [3], [4], inflammatory bowel disease [5], [6], Crohn's disease [6] and bacterial vaginosis (BV) [7], [8], [9], [10].

Use of non-culture based methods to characterize the male urethra in health and disease has lagged behind investigations in other body sites. The hypothesis that male urethral microbiomes could impact STI has been tested using primarily cultivation dependent microbial identification methods [11], [12], [13], [14], [15], [16], [17]. A culture-based study of distal urethral swabs identified diverse bacterial populations in specimens from healthy men, men with chlamydial urethritis, and men with non-chlamydial, non-gonococcal urethritis (NGU) [17]. Another study found that *Lactobacilli spp.* were more frequent in specimens from men who did not have NGU as opposed to NGU positive men [11]. Willen reported that first-catch urine specimens from healthy adult men contained aerobic and anaerobic bacteria, and many of these organisms were absent from corresponding prostrate secretion samples [16]. A study using 16S rRNA allele restriction fragment length polymorphisms (RFLP) identified diverse microbial communities and substantial intra- and inter-person variability in distal urethral swabs from men with urethritis compared to those from healthy men [15]. Thus, it is clear that diverse bacteria commonly colonize the male urethra but there are few data about the normal composition of male urethral microbiomes or if these microbiomes are relevant to male STI.

Here we used 16S rRNA PCR and DNA sequencing to characterize microbial communities in first catch urine, as a proxy for urethral swabs, collected from a cohort of sexually active adult men at high risk for STI. A high degree of inter-subject variability in urine microbiomes was evident but clustering analyses indicated striking differences in the microbiomes of urines from groups of STI positive and negative individuals. In summary, our results indicate that urine from sexually active men often contains complex microbial communities and that the composition of these urine communities is relevant to STI.

## Materials and Methods

### Ethics Statement

All procedures for collection and handling of patient samples and data were approved by the Indiana University-Clarian Institutional Review Board study 0710-80, “Genomic analysis of male urethral micro-environment”. All study subjects provided written informed consent to participate in the study.

### Specimens

Urine specimens were obtained from the Indiana University Infectious Disease Laboratory. Convenience urine specimens were collected from 19 men over the age of 18, at an STD clinic and were frozen at −80°C on the day of collection. Criteria for inclusion in the study were no overt symptoms of urethral infection (e.g. urethral discharge or dysuria) and negative for urethritis (defined as <5 neutrophils per high power field in first catch urine). All men had refrained from taking antibiotics in at least the previous week. Clinical histories and demographic data were available for all subjects and sexual histories were provided by 10/19 (Tables S1 and S2).

### STI testing

Urine specimens were tested for *C. trachomatis* and *N. gonorrhoeae* using the Aptima Combo 2 CT/GC assay (Gen-Probe, San Diego CA), following the manufacturer's instructions. Immediately after removing aliquots for STI testing, residual urine samples were aliquoted and frozen without additives at −80°C.

### DNA isolation

Urine aliquots (up to 50 ml) were thawed and 1.5 ml of each specimen was pelleted by centrifugation at 12,000×g for 10 min at 4°C. DNA was harvested from pellets using a Qiagen DNeasy (Qiagen Inc.) tissue extraction kit using the Gram-positive bacteria protocol, according to the manufacturer's protocol. DNA was stored at 4°C until subsequent 16S PCR amplification and sequencing. Mock specimens (molecular grade water) were processed in parallel with patient samples to monitor reagent purity. Individuals were considered STI positive if Aptima testing revealed pathogens and or if one or more sequences 99% identical to a known urethral pathogen was detected by sequence analysis (*N. gonorrhoeae*, *M. hominis*, *M. genitalium*, *U. urealyticum*.) (Table S3).

### 16S rRNA allele PCR, cloning and sequencing

Urine gDNAs were PCR amplified using the broad range primers 27F 5-AGAGTTTGATCCTGGCTCAG-3′ and 1391R 5′-GACGGGCGGTGWGTRCA-3′ using Takara KA-HS Taq Polymerase (Takara Bio Inc.) in a total reaction volume of 15 µl. PCR cycling conditions were 95°C for 5 min followed by 30 cycles of 95°C for 30s, 55°C for 30s, 72°C for 1.5 min followed by a final cycle of 72°C for 10 min. All urine gDNAs yielded robust amplicons after 30 PCR cycles, whereas identically processed mock specimens (molecular grade water) failed to yield amplicons after 40 cycles. Products from each PCR were gel extracted, tailed with GO TAQ (Promega Inc.) and then TA-cloned into pCRII-TOPO (Invitrogen Inc.). Insert 16S rRNA alleles were sequenced from the ends and middle of the insert using primers M13F 5′-TGTAAAACGACGGCCAGT-3′, M13R 5′-CAGGAAACAGCTATGACC-3 and 907R 5′-CCGTCAATTCCTTTRAGTTT-3′.

The three reads (average length of 769bp) were assembled into a single consensus sequence. 6,877 unique 16S rDNA contigs were assembled with Phrap [18], [19]. Of these, 942 were removed from the subsequent analysis because more than 5% of their average Phred quality scores were <19.

### Bioinformatics and data processing

154 contigs matched human genome sequences, NCBI BLASTn [20] (1) (E-value cutoff 10–20), and were eliminated as likely contaminants. An additional 1,185 contigs were parsed because they failed to pass Bellerophon chimera checking (default parameters) at GreenGenes [21]. The remaining 4,606 contigs (each at least 1,250 bp in length) were considered to represent full length 16S rDNA alleles and were compared to RDP II database [22] sequences using the RDP Classifier program v2.2 [23]. For phylogenetic comparisons of microbiomes, multiple sequence alignments (MLS) of the 16S contig sequences were produced using ClustalW [24] with default parameters, and the MLS were used to construct a neighbor-joining phylogenic tree using PHYLIP [25]. Principal coordinate analyses (PCA) of microbial communities were performed using Unifrac [26], [27]. Hierarchical clustering was performed and heat maps were generated using a Spearman's rank correlation coefficient [28], [29] as a distance measure and a customized script developed in the R statistical package (http://www.r-project.org/). All sequences from this study are available at the Urethral Microbiome Project website (http://www.microbiota.org/cgi-bin/mum/pilot/analysis.cgi).

### Statistics

Chi-square and Fisher-exact tests were used to test if bacterial taxa were biased in urine with or without STI. Wilcoxon rank-sum tests were also performed after reads in each specimen were normalized (i.e., ratios were compared). Pair-wise Spearman's rank correlation coefficients were computed to identify taxa, which were either correlated or anti-correlated in urine specimens. All statistical tests were performed using a customized R script. To correct for multiple tests, false discovery rates were computed with the R package function *q value*.

## Results

### First-catch urine from adult men contains diverse bacterial taxa

We used urines as a proxy for sampling of the male urethra based upon reports that urine and urethral swabs are similarly suitable specimens for detection of diverse urethral pathogens [30], [31], [32], [33]. All 19 urine specimens yielded robust 16S rRNA PCR amplicons following 30 reaction cycles whereas mock control specimens, processed identically to urine, failed to yield amplicons following 40 cycles. All 16S rRNA contigs larger than 1250 bp and Phred quality scores ≥20 were classified using RDP II Classifier Version 2.2 [22], [23]. Total numbers of sequences from individual urine fitting these criteria ranged from 35–352 (SD) 86).

On average, more than 95% of these sequences could be assigned to the genera level using RDP taxonomy with ≥90% confidence and the proportions of sequences in different urines which met this threshold were similar (range 85.8%–98.8%, mean 95.1%, SD 3.6%). The remaining sequences could be classified with 90% confidence to at least the order level, with the exception of 6 Archaeal sequences. Rarefaction analyses, where terminal taxa were classified based on sequence similarity, indicated that collector's curves for most urine samples had entered the plateau phase (Figure S1). Consistent with this, analysis of all sequences from the three specimens which yielded the fewest high quality sequences (U12, U13 and U18) using classifier and a 60% confidence threshold failed to significantly alter the overall composition of the urine communities by Unifrac or to reveal pathogen sequences in the STI negative specimens (U12 and U16) (Data not shown).

### Phylum level composition of urine microbiomes

**Figure 1:**
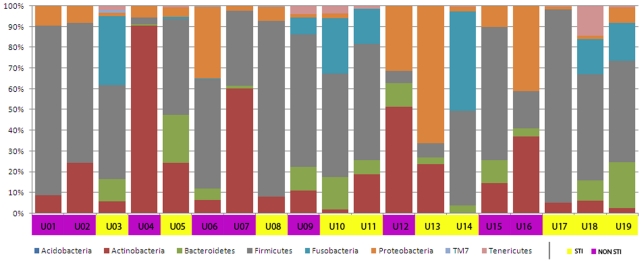
Inter-subject variability in 16S rRNA clones in urine. 4,386-16S rRNA Urine sequences were sorted to the Phyla level using RDP Classifier at 90% confidence cutoff [23]. Subjects are indicated below bars on the X-axis and the percents of clones corresponding to specific phyla are indicated on the Y-axis. STI status of the subjects is indicated below the X-axis.

To identify bacterial taxa, which could represent the “core” urine microbiome 16S rRNA sequences, were classified to the phylum level, according to RDP II taxonomy, and compared. Seven phyla were identified in total. Sequences corresponding to five bacterial phyla including *Firmicutes* (52.6%), *Actinobacteria* (18.7%), *Fusobacteria* (10.0%), *Proteobacteria* (9.4%) and *Bacteroidetes* (7.4%) were frequently detected, whereas sequences corresponding to *Tenericutes* (1.8%) and *TM7* (<0.1%) were less abundant. The distribution of these phyla differed markedly among the urine specimens (Figure 1). *Firmicutes* were present in all specimens and *Actinobacteria* and *Proteobacteria* were each present in 18/19. Proportions of total sequences in urine corresponding to these phyla (where present) ranged from 3.5–93.1%, 0.4–90.3% and 1.3–62.2% for *Firmicute*s, *Actinobacteria* and *Proteobacteria*, respectively. *Bacteroidetes* and *Tenericutes* were identified in 15/19 and 9/19 urine specimens but never constituted more than 25% of total sequences in any urine. In contrast, *Fusobacteria* were only detected in 9/19 urine specimens but accounted for a significant proportion of total sequences (0.4–47.8%, mean 18.9%, SD 15.4%) when present. *TM7* was detected in 3 urine in which it accounted for 0.3–1.5% of the total sequences. These results indicated that there is substantial intra-individual variation in urine microbiomes even at the phylum level.

### Many bacteria in urine are similar to residents of the human vagina, colon and skin

To better characterize the urine microbiomes the 16S rRNA sequences were sorted to the genera level. *Lactobacillus*, *Corynebacterium*, *Streptococcus* and *Sneathia spp.* accounted for approximately 50% of the total urine sequences (Table 1) and the 10 most common genera represented almost 75% of urine sequences, although 72 genera were detected in total (Table S4). This result showed that the overwhelming majority of the urine sequences corresponded to a few abundant genera.

**Table 1:** Abundant urine taxa.

RDP II Identifier	%	NCBI taxa	Source
S001910616	12.6	*Lactobacillus iners*	Urine
S001088824	5.7	Uncultured *Sneathia sp.*	Amniotic fluid
S001546236	5.5	Uncultured *Gemella sp.*	Vagina
S001546274	5.2	Uncultured *Aerococcus sp.*	Vagina
S001550921	4.6	*Corynebacterium sp.*	Urethral swab
S000527990	4.5	*Streptococcus anginosus*	Unknown
S000404352	3.8	*Veillonella montpellierensis*	Blood culture
S001546281	3.7	Uncultured *Prevotella sp.*	Vagina
S001589410	2.8	*Anaerococcus tetradius*	Dental plaque
S001792989	2.0	*Propionibacterium acnes*	Skin
S000391613	1.8	*Atopobium vaginae*	Ovarian abscess
S000128467	1.8	*Corynebacterium tuberculostearicum*	Unknown
S000412018	1.7	*Lactobacillus iners*	Bartholin gland
S000944666	1.7	Uncultured *Streptococcus sp.*	Subgingival plaque
S000944569	1.7	Uncultured *Streptococcus sp.*	Subgingival plaque
S001796262	1.6	*Corynebacterium sp.*	Synovial fluid
S000450504	1.6	*Leptotrichia amnionii*	Female genital tract
S001418339	1.4	*Haemophilus parainfluenzae*	Unknown
S001088822	1.3	Uncultured *Sneathia sp.*	Amniotic fluid
S001744707	1.3	*Streptococcus mitis*	Unknown
S000750307	1.3	*Prevotella timonensis*	Breast abscess
S001744223	1.2	*Mycoplasma hominis*	Urogenital tract
S000965282	1.2	*Delftia acidovorans*	Sewage
S000366404	1.1	*Corynebacterium pseudogenitalium*	Urogenital tract
S001907903	1.0	*Clostridiales oral taxon C16*	Oral cavity

Urine 16S rRNA sequences were blasted against the RDP database to identify their closest match. The percent column indicates the percent of total urine sequences, which most closely match the specific RDP taxa. The column labeled source indicates the type of sample from which the RDP sequence was isolated, if known.

The most similar sequence in the RDP database corresponding to each urine sequence and the original source of the RDP sequence were identified. More than 72% of the urine sequences best-matched only 25 RDP sequences. Most of these RDP sequences were originally from human female urogenital tract specimens [7], [9], [34], [35] and smaller proportions were from the mouth and skin (Table 1) [36]. The most common urine sequences corresponded to *Lactobacillus iners*. Other abundant taxa included various species of *Aerococcus*, *Anaerococcus*, *Prevotella*, *Gemella*, *Veillonella* and *Sneathia* (Table 1 and Table S5).

### Clustering analysis can separate STI and non-STI urine microbiomes

**Figure 2:**
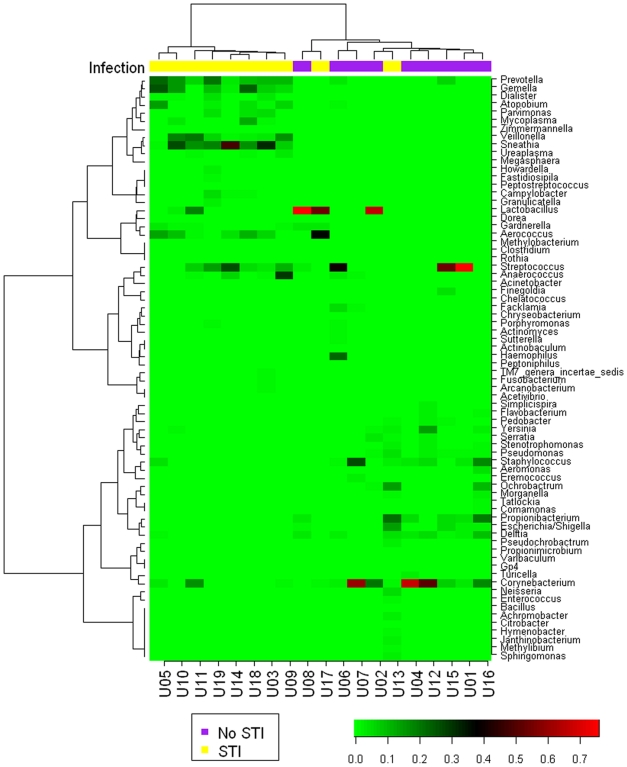
Hierarchical clustering of urine microbiomes. Genus names corresponding to terminal taxa depicted in the heat map are listed to the right of the figure. Subjects are listed at the bottom. Intensity of the coloration of cells in the heat map indicates the extent of correlation of different urine samples. Dendograms at the top and right of the heat map indicate relationships of microbiomes and genera, respectively. The top infection color bar indicates STI (labeled as YES) or non-STI (labeled as NONE) participants.

To identify potential associations between bacterial taxa the urine microbiomes were hierarchically clustered and genera were weighted to reflect their abundance. Figure 2 depicts clustering results produced using pair-wise Spearman's rank correlation coefficients among all the genera from urine. The first cluster (Fig. 2, from the left end) contains eight urine specimens, all of which were positive for one or more STI by specific nucleic acid amplification tests and/or by the presence of sequences corresponding to known sexually transmitted pathogens (Table S3). All eight specimens in this cluster contained *Prevotella* and *Sneathia* spp., *Prevotella* was only detected in 2 specimens outside this group, and *Sneathia spp.* were unique to this group. Other frequent and or abundant genera in this group included *Dialister* (7/8), *Gemella* (7/8), and *Atopobium* (5/8). The second cluster contained two specimens and the majority of sequences in both of these were *Lactobacillus iners*. One of these was also positive for *Mycoplasma spp.* There are nine specimens in the third cluster. Although there was substantial diversity comparing these urine, one or more of *Corynebacteria*, *Propionobacteria*, *Staphylococcus* and or *Streptococci spp.* were dominant. Approximately 5% of the sequences from one urine (Urine 13) corresponded to a reference strain of *N. gonorrhoeae* (>99% identical), but this was the only STI positive urine in this cluster (Table S3).

**Figure 3:**
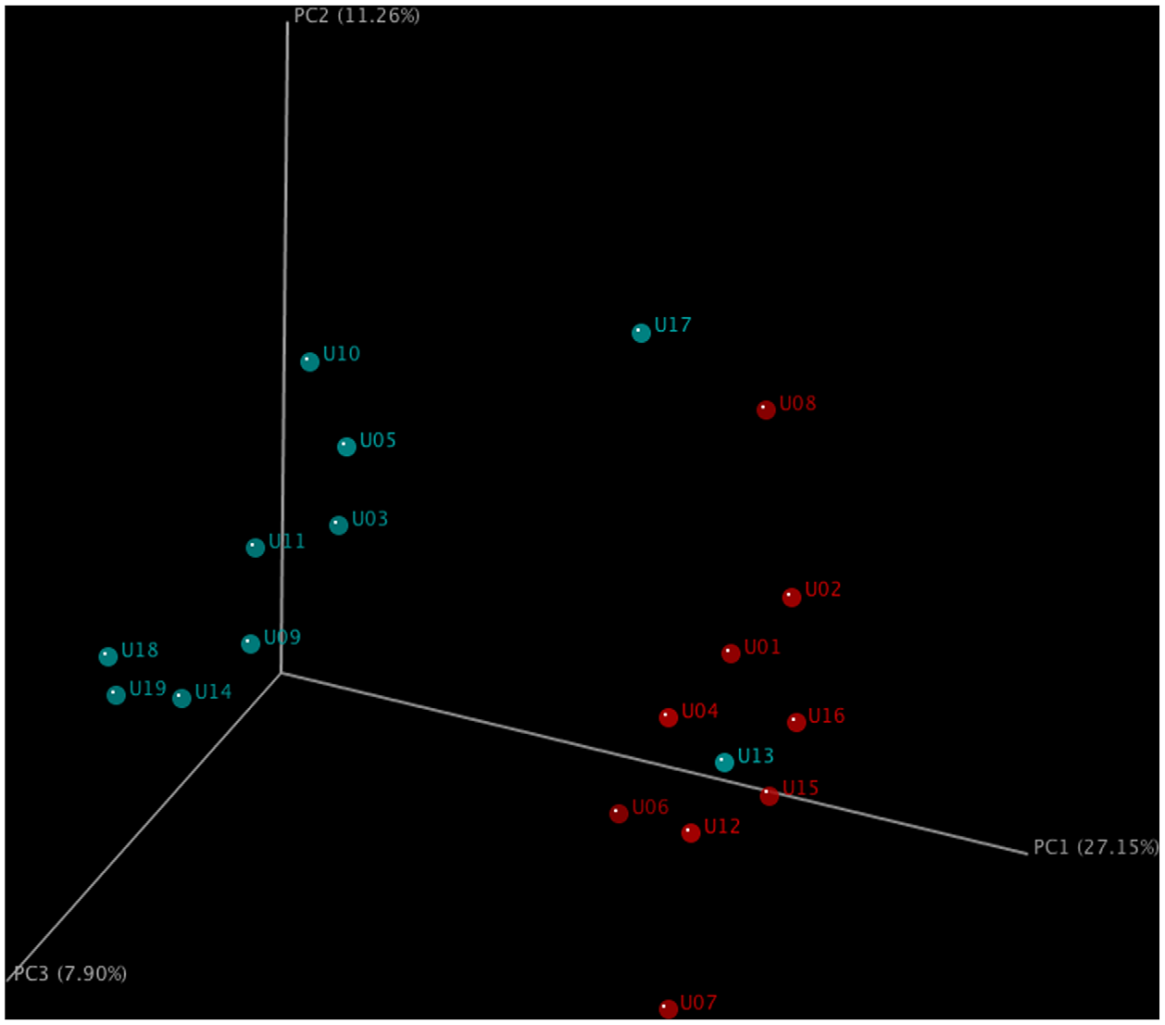
Unifrac community comparison of urine. Clustering of urine microbiomes was performed using un-weighted Unifrac [37]. The percent variations explained by each principle component are indicated on the axis. Blue circles indicate urine from individuals positive for STI; red circles correspond to urine negative for STI.

To independently assess associations between STI and specific microbiomes the data were analyzed by un-weighted Unifrac PCA [26], [37]. This analysis provides a quantitative measure of β-diversity which reflects the evolutionary distance between the component taxa in the communities [38]. Unifrac reproduced the groups revealed by hierarchical clustering (Figure 3). Specifically, all eight specimens from cluster 1 grouped together and separately from specimens in clusters 2 and 3.

To identify organisms driving the cluster results, genera that were significantly enriched in urines from men with STI were identified using Fisher's exact, Chi-square, and Wilcoxons tests (Table S6). Many of the abundant genera in cluster 3 were enriched in men with STI. To detect if associations between any urine taxa existed, Spearman's rank correlation coefficients were calculated for all pair-wise combinations of the genera identified in this study (Table S7). The abundant genera in cluster one discussed above were strongly correlated with one another and anti-correlated with abundant genera from the other clusters. For example, *Sneathia* was strongly positively correlated with *Gemella*, *Dialister*, *Aerococcus*, *Prevotella* and *Veillonella*, and negatively correlated with *Staphylococcus*, *Propionibacterium* and *Streptococcus*.

## Discussion

We report a detailed cultivation independent comparison of urine microbiomes in men with and without STI. Large-scale Sanger sequencing of near full-length 16S rRNA alleles was utilized so the data set would be a robust resource for future comparisons.

Our data support an expanded view of the association of the urogenital microbial communities in health and disease. First, the bacterial communities in urine are complex and a single, characteristic microbial community was not apparent. Urogenital genera overlap to some degree microbial communities found in the superficial skin [36], colon [39], vagina [7], [9], [34], [35]. A variety of taxa identified on the penile coronal sulcus in a recent study were also observed [40]. However, our data suggest the overall composition of urine and coronal sulcus microbiomes differ. The sulcus is dominated by *Pseudomonadaceae spp.* [40] whereas sequences corresponding to this family were rare in urine. Second multiple BV-associated anaerobic taxa, such as *Sneathia spp.*, which appear to be minor components of sulcus microbiomes in uncircumcised men were dominant components of urine microbiomes. Finally, *Lactobacillus* spp. were abundant in some urines but were not reported in sulcus specimens. Caveats of these comparisons include subject populations (African men versus an ethnically diverse population of American men). The power of our study was also too low to assess differences in microbiomes associated with race or ethnicity. Finally this study and the survey in question employed different PCR primers, PCR amplification conditions and 16S rRNA sequencing, all of which limit the utility of direct comparisons of the data sets.

A second key finding is the association of STI with a cluster of organisms not previously linked to the male urogenital tract: *Sneathia*, *Gemella*, *Aerococcus*, *Anaerococcus*, *Prevotella* and *Veillonella*, to name a few [9], [35], [41]. These organisms have previously been identified in the coronal sulcus of uncircumcised men, and became less frequent following circumcision to reduce risk of sexually transmitted human immunodeficiency virus infection [40]. Some of these organisms are also commonly observed in the vaginal microbial communities of asymptomatic women [7], as well as women with bacterial vaginosis [9], [41] and upper genital tract pathology [42], [43]. Similar anaerobic communities in female genital tract are associated with increased risk for STI [44]. These observations suggest the hypothesis that establishment of BV-like communities in the male urethra is similarly associated with STI risk. However, present data are clearly inadequate to differentiate if the STI-associated communities precede, are co-transmitted with or are established subsequent to STI.

An limitation of this study is that urine can sample multiple body compartments and that these specimens were used as a proxy for urethral swabs because the latter technique is contraindicated in STI negative men. Urethral swabs and urine are equally suitable specimens for PCR-based detection of a variety of sexually transmitted pathogens [30], [31], [32], [33]. Comparisons of paired first-catch urine and urethral swabs support that interpretation that the microbiomes sampled by with these specimen types are highly similar (Q Dong *et al.*, manuscript in preparation). Thus we assume here that urine specimens primarily contain bacteria associated with the urethral epithelium, and lower numbers of organisms from the bladder and urethral meatus. Incidence of upper urinary tract infection is low in our study population which suggests bladder flora were infrequent. Thus, although male urine may not exclusively sample urethral flora, we believe it provides as good of a measure of this flora as is practical and ethical in individuals not suspected to have STI.

In conclusion, our data provide the first glimpse into the urogenital microbiome in men with STI. Caution is clearly warranted in interpreting associations between characteristic microbiomes and STI; we cannot exclude other possible explanations for the patterns observed, such as long-term effects of undocumented antibiotic use [45]. Considering these and similar caveats we believe the data suggest the hypothesis that establishment characteristic microbiomes might be related to risk for subsequent STI. Longitudinal studies of urine microbiomes prior to and post STI will be necessary to test this and we believe such studies could elucidate inter-species microbial interactions relevant to a variety of poorly understood urogenital syndromes.

## Supporting Information

Table S1 Subject characteristics.
(0.07 MB DOC)

Table S2 Demographics and recent sexual history.
(0.08 MB DOC)

Table S3 Sequences corresponding to pathogens.
(0.11 MB DOC)

Table S4 Complete list of urine genera sorted by RDP classifier.
(0.03 MB XLS)

Table S5 Complete list of urine taxa by RDP best match.
(0.48 MB XLS)

Table S6 Statistical comparisons of correlated urine taxa.
(0.03 MB XLS)

Table S7 Spearman's rank correlation coefficients for all urine taxa.
(0.61 MB XLS)

Figure S1 Rarefaction curves of individual urine microbiomes. Rarefaction curves were generated using the MOTHUR package after clustering sequences at the genetic distance cutoffs of 0.03, 0.05, and 0.10.
(0.48 MB PDF)
