# Supplementary material for: Characteristic Male Urine Microbiomes Associate with Asymptomatic Sexually Transmitted Infection
Source: PLoS One. 2010 Nov 24;5(11):e14116. doi: 10.1371/journal.pone.0014116 (PMC2991352; doi:10.1371/journal.pone.0014116)
Supplement: Table S1 — Subject characteristics. (0.07 MB DOC) [file pone.0014116.s001.doc]

| **Supplemental table 1. Subject Characteristics** | | |
| --- | --- | --- |
|  | Clinician | Fast-track |
| Age^1^ | 32.1 (21-54) | 32.6 (21-52) |
| Black | 70% | 40% |
| *Chlamydia trachomatis^2^* | 1 | 0 |
| *Neisseria gonnorhea*^3^ | 1 | 1 |
| *Ureaplasma spp*. | 4 | 2 |
| *Mycoplasma spp*. | 5 | 1 |
| Last Sexual Exposure^1^ | 9.1 (0-30) | NA |
| Vaginal^4^ | 9 | NA |
| Oral^4^ | 9 | NA |
| Anal^4^ | 1 | NA |
| 1. Age and days since last sexual exposure is given as averages and ranges (parenthesis) in years and days, respectively. 2. *C. trachomatis* infection was diagnosed by Aptima, the broad range primers used in this study lack homology to the *C. trachomatis* 16S rRNA allele. 3. U13 listed as *N. gonorrhoeae* + tested negative by Amplicor, but *N. gonorrhea* DNA was detected by 16S rRNA sequencing. 4. Sexual exposure type, Vaginal = penile-vaginal; Oral = penile-oral; Anal = penile-anal. | | |
