# Supplementary material for: Characteristic Male Urine Microbiomes Associate with Asymptomatic Sexually Transmitted Infection
Source: PLoS One. 2010 Nov 24;5(11):e14116. doi: 10.1371/journal.pone.0014116 (PMC2991352; doi:10.1371/journal.pone.0014116)
Supplement: Table S2 — Demographics and recent sexual history. (0.08 MB DOC) [file pone.0014116.s002.doc]

| **Supplemental table 2: Demographics and recent sexual history** | | | | | | |
| --- | --- | --- | --- | --- | --- | --- |
| Patient | Age | Race | Vaginal | Oral | Anal | DLSE |
| U05 | 23 | C | Y | Y | N | 0 |
| U06 | 34 | L | Y | Y | N | 1 |
| U07 | 35 | B | Y | Y | N | 0 |
| U08 | 22 | B | Y | Y | N |  |
| U09 | 27 | C | N | Y | Y | 13 |
| U10 | 30 | B | Y | N | N | 12 |
| U14 | 21 | B | Y | Y | N | 30 |
| U17 | 32 | B | Y | Y | N | 9 |
| U18 | 43 | B | Y | Y | N | 7 |
| U19 | 54 | B | Y | Y | N | 10 |
| U01 | 21 | O |  |  |  |  |
| U02 | 32 | B |  |  |  |  |
| U03 | 24 | C |  |  |  |  |
| U04 | 52 | C |  |  |  |  |
| U11 | 26 | C |  |  |  |  |
| U12 | 50 | B |  |  |  |  |
| U13 | 33 | B |  |  |  |  |
| U15 | 36 | C |  |  |  |  |
| U16 | 29 | C |  |  |  |  |
| Race = self-identified race. B, black; C; Caucasian; L, Latino; O, Other. Sexual activities, Y = yes, N = no, Blank = no data. Vaginal = penile-vaginal; Oral = penile-oral; Anal = penile-anal. DLSE = days since last sexual exposure. | | | | | | |
