# Supplementary material for: Characteristic Male Urine Microbiomes Associate with Asymptomatic Sexually Transmitted Infection
Source: PLoS One. 2010 Nov 24;5(11):e14116. doi: 10.1371/journal.pone.0014116 (PMC2991352; doi:10.1371/journal.pone.0014116)
Supplement: Table S3 — Sequences corresponding to pathogens. (0.11 MB DOC) [file pone.0014116.s003.doc]

| **Supplemental table 3: Sequences corresponding to pathogens** | | | | | | | |
| --- | --- | --- | --- | --- | --- | --- | --- |
| Patient | Total Sequences | HQ Sequences | AP-NG | AP-CT | HQ Sequences*  NG M U | | |
| U01 | 345 | 115 |  |  |  |  |  |
| U02 | 375 | 291 |  |  |  |  |  |
| U03 | 372 | 275 |  |  |  | 4 | 1 |
| U04 | 358 | 258 |  |  |  |  |  |
| U05 | 377 | 253 |  | Pos |  | 2 |  |
| U06 | 376 | 259 |  |  |  |  |  |
| U07 | 381 | 282 |  |  |  |  |  |
| U08 | 374 | 333 |  |  |  |  |  |
| U09 | 365 | 352 |  |  |  |  | 14 |
| U10 | 344 | 273 |  |  |  | 9 | 1 |
| U11 | 291 | 175 |  |  |  |  | 2 |
| U12 | 375 | 35 |  |  |  |  |  |
| U13 | 346 | 207 |  |  | 11 |  |  |
| U14 | 376 | 276 |  |  |  |  | 1 |
| U15 | 351 | 269 |  |  |  |  |  |
| U16 | 374 | 73 |  |  |  |  |  |
| U17 | 369 | 317 |  |  |  | 1 |  |
| U18 | 364 | 310 | Pos |  |  | 39 | 5 |
| U19 | 345 | 253 |  |  |  | 2 |  |
| Total sequences indicate number of unique clones sequenced from patient specimen. HQ (High quality) sequences indicate sequences remaining following quality filtering. AP-NG and AP-CT indicate Aptima combo test results for *N. gonorrhoeae* and *C. trachomatis*, respectively. Sub-columns under HQ Sequences at right correspond to the number of sequences 99% or more identical to *N. gonorrhoeae* (NG) or *Mycoplasma* (M) and *Ureaplasma* (U) species that are known urethral pathogens. | | | | | | | |
