# Supplementary material for: Characteristic Male Urine Microbiomes Associate with Asymptomatic Sexually Transmitted Infection
Source: PLoS One. 2010 Nov 24;5(11):e14116. doi: 10.1371/journal.pone.0014116 (PMC2991352; doi:10.1371/journal.pone.0014116)
Supplement: Figure S1 — Rarefaction curves of individual urine microbiomes. Rarefaction curves were generated using the MOTHUR package after clustering sequences at the genetic distance cutoffs of 0.03, 0.05, and 0.10. (0.48 MB PDF) [file pone.0014116.s008.pdf]

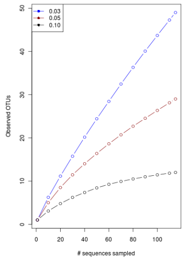

Sample 1

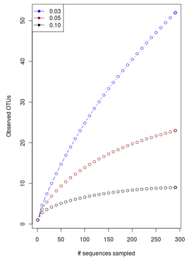

Sample 2

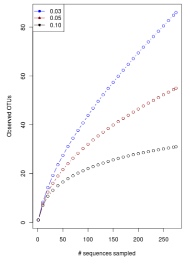

Sample 3

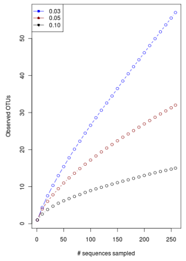

Sample 4

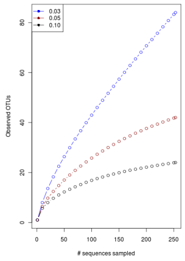

Sample 5

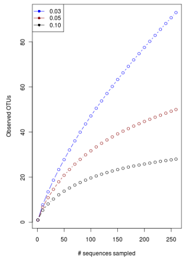

Sample 6

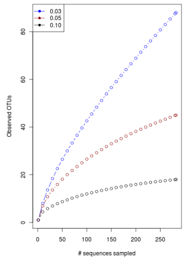

Sample 7

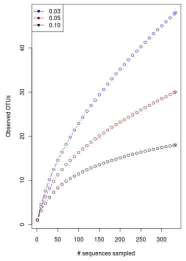

Sample 8

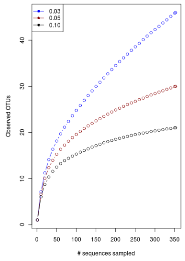

Sample 9

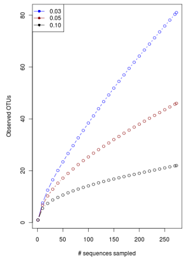

Sample 10

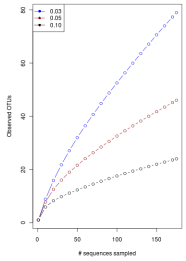

Sample 11

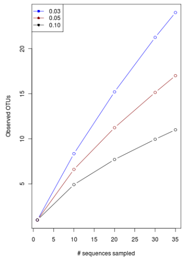

Sample 12

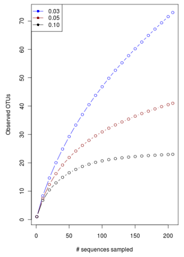

Sample 13

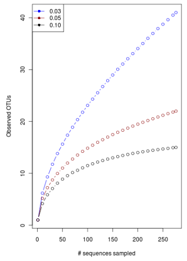

Sample 14

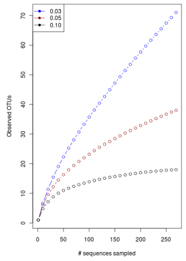

Sample 15

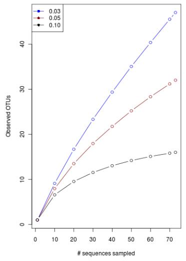

Sample 16

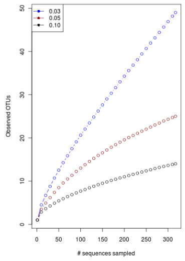

Sample 17

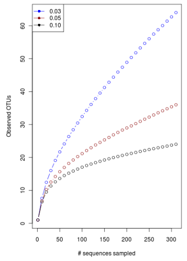

Sample 18

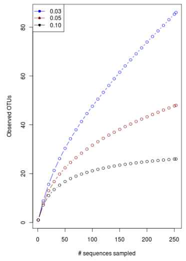

Sample 19
